# First molecular detection and genetic characterization of porcine circovirus 4 in the Gansu Province of China

**DOI:** 10.1371/journal.pone.0293135

**Published:** 2024-02-05

**Authors:** Peng-Fei Fu, Yan-Hong Wang, Guo Liu, Dong-Mei Wang, Wei-Wei Huang, Duan-Qiang Guo, Xin-Yang Li, Ping Liu, Meng-Xiang Wei, Min Lu, Jun Hong

**Affiliations:** 1 College of Life Science and Engineering, Henan University of Urban Construction, Pingdingshan, Henan Province, China; 2 College of Veterinary Medicine, Gansu Agricultural University, Lanzhou, Gansu Province, China; Shanxi University, CHINA

## Abstract

Since its initial discovery in the Hunan province of China, genomic DNA of porcine circovirus 4 (PCV4) has been detected in pigs across multiple provinces in China, as well as in South Korea. However, the prevalence of porcine circovirus type 4 in Gansu Province, China, remains unknown. To address this gap, we undertook an extensive study where we gathered 121 clinical samples displaying diverse clinical manifestations from pig farms in Gansu Province between 2022 and 2023. Employing a real-time fluorescence quantification method, we identified the presence of PCV4 genome. Out of the 121 clinical samples analyzed, 13 samples tested positive for PCV4, resulting in a positive rate of 10.74% (13/121). This finding confirms the presence of PCV4 in pig farms within Gansu Province, China. Furthermore, we successfully sequenced and analyzed the complete genomes of two distinct PCV4 strains, comparing them with 60 reference sequences archived in the GenBank database. The results revealed a high nucleotide homology (98.2–98.8%) between the strains obtained in this study and the PCV4 reference strains, indicating a relatively low evolutionary rate of the PCV4 genome. Phylogenetic analysis revealed that two strains in this study belong to PCV4a and PCV4c. As far as we know, this study marks the inaugural report on the molecular identification and genomic attributes of PCV4 in Gansu Province, China, offering valuable insights for devising preventive and control strategies against this emerging virus.

## Introduction

Porcine circoviruses (PCVs) are members of the *Circovirus* genus within the *Circoviridae* family [1–3]. These viruses are characterized by their distinctive single-stranded circular DNA configuration, enclosed within an icosahedral virion measuring roughly 17 nm in diameter [3]. Up to now, four PCVs with similar structures have been recognized: porcine circovirus 1 (PCV1), porcine circovirus 2 (PCV2), porcine circovirus 3 (PCV3), and porcine circovirus 4 (PCV4). The genomes of PCVs consist of circular, single-stranded DNA molecules, with sizes ranging from approximately 1.7 to 2.1 kb. [1–3].

PCV1 was initially documented in 1982 and is generally considered non-pathogenic to pigs [4–6]. In the 1990s, PCV2 was discovered, and it was associated with various clinical conditions, including postweaning multisystemic wasting syndrome (PMWS), porcine dermatitis and nephrotic syndrome (PDNS), as well as reproductive and respiratory disorders [7–11]. These conditions have resulted in substantial economic losses for the global pig industry [7–11]. The disease associated with PCV2 is now commonly summarized as porcine circovirus (related) disease (PCVD/PCVAD) [7–11]. PCV3 was identified in 2015 using next-generation sequencing (NGS) methods in pigs with clinical conditions such as PDNS, reproductive failure, myocarditis, or multisystemic inflammation [12, 13]. Porcine circovirus 4 (PCV4) was first discovered in 2019 in Hunan Province of China, as a novel species of circovirus [14]. The genome of PCV4 could also be found in healthy pigs, in spite of pigs with severe clinical signs including respiratory disease, enteritis and PDNS [15]. Currently, PCV4 has been documented in various provinces in both China and Korea [16–20]. However, it’s worth noting that PCV4 DNA hasn’t been found in samples collected from pigs in Europe (specifically Italy and Spain) and South America (Colombia) [21, 22]. In addition, PCV4 was successfully rescued from an infectious clone and proved to be pathogenic to piglets [23], indicating it as a newly discovered virus was a potential threat to the pig industry.

The aim of this study was to assess the occurrence of PCV4 in pig farms situated in Gansu, China, during the period from 2022 to 2023. Furthermore, complete genomes were amplified to explore the genetic variability of the virus present in Gansu Province.

## Material and methods

### Samples collection and viral genome extraction

A total of 121 samples were collected from 14 pig farms in 8 cities (Jinchang, Wuwei, Baiyin, Lanzhou, Dingxi, Gannan, Tianshui and Pingliang) during 2022–2023 (Table 1). Among the collected samples, 89.26% (108/121) were obtained from diseased pigs exhibiting various clinical signs, including respiratory, enteric, and PDNS, for diagnostic purposes. The remaining 10.74% (13/121) were collected from healthy animals for infectious disease surveillance. The diseased pigs were humanly euthanized with an overdose of pentobarbitalum natricum [Natriumpentobarbital 20%^®^ 40 mg/kg iv in the V. jugularis externa]. Then, samples were collected. In healthy pigs, venous blood and swab paper were collected after intramuscular injection of ketamine 15 mg/kg. The sample types encompassed brain, kidney, spleen, lung, serum, oral fluid, feces, lymph node, tonsil, nasal swab, placenta, intestine, and semen.

**Table 1:** Geographic distribution of the 121 samples and PCV4 positivity rates in these cities.

Geographical location	Number of samples	PCV4
Jinchang	11	0/11
Wuwei	26	4/26
Baiyin	19	5/19
Lanzhou	21	4/21
Dingxi	13	0/13
Gannan	14	0/14
Tianshui	9	0/9
Pingliang	8	0/8
Total	121	13/121

All experimental procedures were reviewed and approved by the Henan University of Urban Construction Animal Care and Use Committee (license number HNXK (Henan) 2015–0001). All samples were collected under the supervision of veterinarians from College of Life Science and Engineering. All efforts were made to minimize their suffering throughout the experiment.

Each sample was individually processed by mixing it with phosphate-buffered saline (PBS) in a sterile 1.5-mL microcentrifuge tube. Subsequently, it underwent three cycles of freezing and thawing before being centrifuged at 12,000 ×g. The resulting supernatant was either utilized for immediate viral genome extraction or stored at -80°C until further use. DNA and RNA extraction procedures were carried out using the FastPure Viral DNA/RNA Mini Kit from Vazyme Biotech Co., Ltd., Nanjing, China, in accordance with the manufacturer’s guidelines. The viral genome was then identified through a real-time PCR assay based on SYBR Green І, as detailed in a previous publication [19]. Meanwhile, other common viruses, including porcine epidemic diarrhea virus (PEDV), porcine reproductive and respiratory syndrome virus (PRRSV), Porcine deltacoronavirus (PDCoV), swine acute diarrhea syndrome-coronavirus (SADS-CoV) pseudorabies virus (PRV), porcine circovirus 2 (PCV2) and porcine circovirus 3 (PCV3) were also detected using PCR or RT-PCR assay as previously described [24–29].

### Complete genome sequencing

As described previously [30], the complete genome of PCV4 was sequenced employing three sets of overlapping primers (S1 Table). The PCR mixture was composed of 10 μL of KOD OneTM PCR Master Mix (Toyobo (Shanghai) Biotechnology Co., Ltd., China), 0.5 μL each of forward and reverse primers (at 10 mM concentration), 1 μL of template DNA, and 8 μL of double-distilled water. The amplification parameters for these three primer pairs were as follows: 98°C for 30 s; 30 cycles of 98 °C for 10 s, 60°C for 5 s, and 68°C for 5 s. According to the manufacturer’s instructions, the PCR amplification products were purified using MagExtractor^™^ -PCR & Gel Clean up- (Toyobo, Shanghai, China) and cloned into the pMD18-T vector (Takara, Dalian, China) for constructing recombinant plasmid. The recombinant plasmids propagated in *Escherichia coli* DH-5α cells (Tolo Biotechnology, Shanghai, China). The confirmed positive clones were dispatched to Sangon Biotech Co., Ltd (Shanghai, China), for sequencing. The complete genome was then assembled utilizing the EditSeq and Megalign programs within the LaserGene software package (DNASTAR, Inc., Madison, WI).

### Sequence alignment and phylogenetic analysis

The complete genomes of PCV4 strains in this investigation underwent analysis alongside 60 reference strains cataloged in GenBank. Information regarding the reference strains was summarized in S2 Table. Nucleotide and deduced amino acid sequences were aligned using the Clustal W method in the MegAlign program. Molecular Evolutionary Genetics Analysis (MEGA) software (version 7.0) was applied to construct phylogenetic tree by the neighbour‐joining method with a p-distance model, and a bootstrap of 1000 replicates.

## Results and discussion

Previous studies have shown that porcine circovirus is prevalent in swine production worldwide. PCVAD caused by PCVs, especially PCV2 and PCV3, has caused significant economic losses to the global pig industry. Therefore, PCV4 has attracted a lot of attention as an emerging porcine circovirus in 2019. Moreover, a recent study suggests that PCV4 is pathogenic to piglets [23]. However, information on the prevalence of PCV4 in Gansu Province, China is lacking. Therefore, to fill this gap, this study conducted an epidemiologic survey of PCV4 in Gansu Province.

Currently, there are various molecular epidemiology studies on PCV4 in pigs across certain provinces of China and Korea, reporting positivity rates ranging from 1.6% to 45.39% [31, 32]. In this study, 121 samples were collected from 121 pigs with different clinical manifestations in Gansu Province, China. Of these, PCV4 was identified in 10.74% (13/121) of samples, which was far lower than that of PCV4 45.39% (69/152) in Henan Province reported by Hou et al but higher than that of PCV4 1.6% (27/1683) in Inner Mongolia Province [31, 32]. Details of the 13 positive samples were summarized in Table 2. Of the 13 positive samples, 38.46% (5/13) originated from kidneys, 23.08% (3/13) from intestines, 23.08% (3/13) from lungs, and 15.38% (2/13) from oral fluids. Other studies have demonstrated the presence of PCV4 DNA in nearly all tissues, including sera, heart, liver, spleen, lung, kidney, lymph nodes, tonsils, intestines, and brain, in both diseased piglets and healthy animals [31–33]. These results indicate that PCV4 has a broad tissue propensity to facilitate horizontal and vertical transmission. The clinical samples were collected from 8 cities during 2022–2023. PCV4 was identified from samples of 3 cities (Lanzhou, Wuwei, Baiyin) (Table 1). PCV4 was not detected in other cities, possibly due to the limited number of samples available. The 13 positive samples came from 13 diseased pigs at different stages of growth, presenting symptoms such as respiratory disease, diarrhea, and PDNS. These findings suggested that PCV4 DNA could be detected in Gansu Province with a low positive rate and limited geographical distribution. In my opinion, prompt measures should be implemented to mitigate the potential threat posed by PCV4 to the pig industry in Gansu Province.

**Table 2:** Origin, clinical manifestation and detection results of 13 PCV4 positive clinical samples from the Gansu Province of China during 2022–2023.

Sample name	Collection date	Sample type	Geographical location	Farm	Growth stages	Clinical symptoms	PEDV	TGEV	PDCoV	SADS-CoV	PRV	PCV2	PCV3	PRRSV
Sample 1*	2022.10.16	intestine	Baiyin	Farm A	newborn	diarrhoea	+	-	-	-	-	-	-	-
Sample 2	2022.10.16	intestine	Baiyin	Farm A	newborn	diarrhoea	+	-	-	-	-	-	-	-
Sample 3	2022.10.16	intestine	Baiyin	Farm A	newborn	diarrhoea	+	-	-	-	-	-	-	-
Sample 4	2022.11.10	lung	Wuwei	Farm B	grower	respiratory disease	-	-	-	-	-	-	-	+
Sample 5	2022.11.10	lung	Wuwei	Farm B	finisher	respiratory disease	-	-	-	-	-	-	-	+
Sample 6	2022.12.03	kidney	Lanzhou	Farm C	grower	PDNS	-	-	-	-		+	-	-
Sample 7*	2022.12.03	kidney	Lanzhou	Farm C	grower	PDNS	-	-	-	-		+	-	-
Sample 8	2022.12.18	kidney	Wuwei	Farm D	grower	PDNS	-	-	-	-		+	+	-
Sample 9	2022.01.18	kidney	Baiyin	Farm E	piglet	PDNS	-	-	-	-			+	-
Sample 10	2023.01.18	kidney	Baiyin	Farm E	piglet	PDNS	-	-	-	-			+	-
Sample 11	2023.01.30	oral fluids	Lanzhou	Farm F	weaning	No	-	-	-	-			-	-
Sample 12	2023.01.30	oral fluids	Lanzhou	Farm F	weaning	No	-	-	-	-			-	-
Sample 13	2023.02.10	lung	Wuwei	Farm G	finisher	respiratory disease	-	-	-	-			-	-

Note:

The complete genomes of GS2022-BY and GS2022-LZ were acquired from sample 1 and 7 marked with *. No represents no obvious clinical manifestations.

Besides the infection of PCV4 described above, we investigated the coinfection of PCV4 with other pathogens, such as PCV2, TGEV, SADS-CoV, PCV3, PRV, PDCoV, PEDV and PRRSV. In brief, 23.08% (3/13) of PCV4-positive samples were coinfected with PCV2, 23.08% (3/13) were coinfected with PCV3, 15.38% (2/13) were coinfected with PRRSV and 23.08% (3/13) were coinfected with PEDV. Interestingly, one sample was positive for PCV2, PCV3 and PCV4. The remaining 3 samples were PCV4 positive with no other pathogens detected, and two of which did not show clinically obvious signs. These samples were submitted for diagnosis. Unfortunately, due to the fact that the tissue samples were not fixed with 4% paraformaldehyde, we were unable to perform hematoxylin and eosin (H&E) staining and immunohistochemical assays. Consequently, we could not investigate the potential association between PCV4 and pathological changes. Currently, we are making efforts to isolate PCV4 strains. If successful, animal return experiments will compensate for these regrets.

To gain insights into the genetic characteristics of PCV4 in Gansu Province, we successfully obtained and deposited two distinct complete genomes (GS2022-BY and GS2022-LZ) in the GenBank database. These genomes have been assigned the accession numbers OQ970016 and OQ970017, respectively. The reasons why only two strains have been sequenced were as follows: First, the viral load of some samples was low. Second, the genome of PCV4 has a stem-loop structure that makes it difficult to amplify. Compared to the reference strains, no base insertions and deletions occurred in the whole genomes of GS2022-BY and GS2022-LZ with a size of 1770 nt. Then, GS2022-BY and GS2022-LZ were analyzed with 60 reference strains deposited in GenBank (S2 Table). Sequence analysis indicated that the GS2022-BY and GS2022-LZ shared 98.6% whole genome nucleotide identity, and these two strains showed high identities of 97.9–99.5% to the genomes of the reference strains. These reference strains were detected in different species (pigs, dogs, foxes and cows) and in different geographical distributions (several provinces in China and Korea) [30, 34–36]. The genomes of these reference strains date back to as early as 2012 [32]. The high homology observed between the strains in this study and reference strains from various sources suggests that the PCV4 genome is highly conserved.

**Fig 1:**
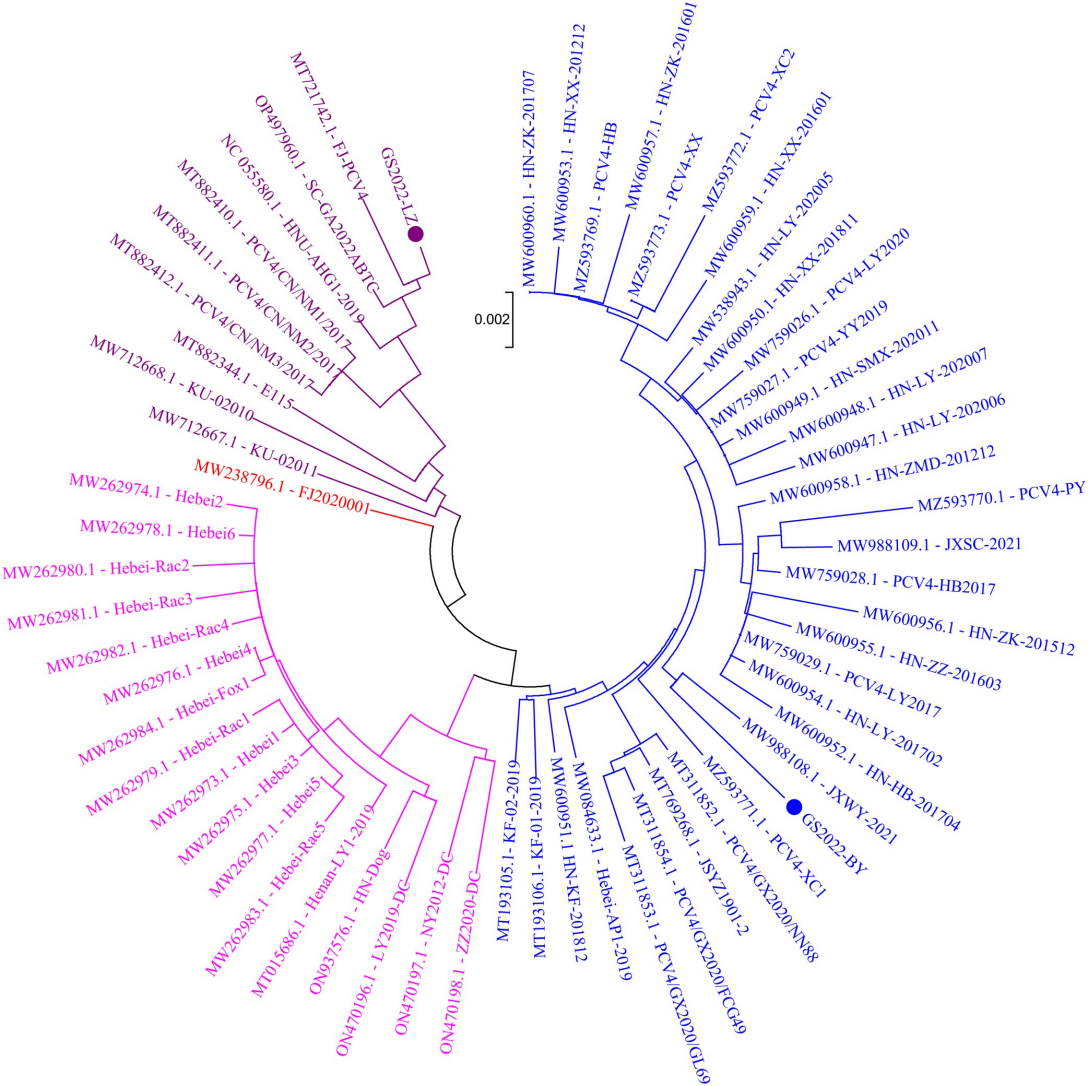
The neighbor-joining tree was constructed based on the complete genome of 62 PCV4 strains with a p-distance model and bootstrapping at 1,000 replicates. Different colors represent different genotypes. Blue, fuchsia and purple represent the three genotypes of PCV4 (PCV4a, PCV4b and PCV4c) proposed by Xu et al (2022), respectively, while red represents undefined genotype.

The limited number of existing reference strains and the high genomic identity between these strains pose challenges in classification of PCV4. Nevertheless, based on several suggested classification schemes [19, 30], PCV4 has been categorized into either two (PCV4a and PCV4b) or three primary clades (PCV4a, PCV4b, and PCV4c). As previously described, we constructed a NJ phylogenetic tree (Fig 1) [30], keeping all parameters the same except for an expanded set of reference strains. According to the proposed clade classification and amino acid marker positions outlined by Xu et al. [30], three different genotypes, namely PCV4a, PCV4b and PCV4c were observed in Fig 1, and the two strains in this study clustered in the proposed PCV4a and PCV4c, respectively. Curiously, the phylogenetic tree suggested that a PCV4 reference strain belonged to PCV4c in Xu et al, whereas in this study it was included in an undefined cluster between PCV4b and PCV4c. Meanwhile, it is difficult to elucidate any potential clade distribution by geographic location, as PCV4 sequences found on different farms in several provinces in China and Korea were represented in more than one clade, while sequences coming from another part of the provinces (e.g. Inner Mongolia) were found in a single group. These observations suggested that the genotype classification proposed by Xu et al. (2022) might have limitations. It is recommended to prioritize genetic distance and phylogenetic clustering as the primary criteria [37]. Additionally, other factors such as the number of sequences within clusters, host and geographic clustering, concordance between different genomic regions, and analysis methods should be considered to develop an effective classification system for research and diagnosis [37]. Therefore, the genotype classification scheme of PCV4 warrants further study.

**Fig 2:**
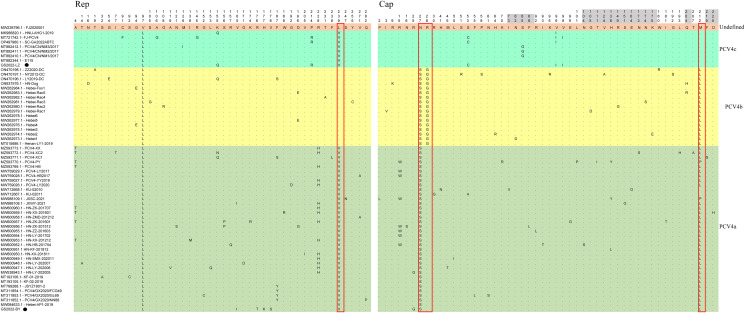
All amino acid mutations in Rep and Cap. Different background colors indicate different genotypes proposed by Xu et al (2022), respectively. The red open box showed the amino acid markers of genotypes proposed by Xu et al (2022). The amino acid positions in the gray region were included in the potential linear B-cell epitopes predicted by Wang et al (2021). The PCV4 strain in this study was marked by solid circles.

There were no amino acid insertions and deletions in the Rep and Cap proteins of these 62 strains. All identified amino acid mutations in Rep and Cap of the 62 PCV4 strains were depicted in Fig 2. The proposed marker positions determining clade divisions were located in the amino acid sites V239L for Rep and, N27S, R28G and M212L for Cap [30]. Concisely, PCV4a contains an amino acid pattern (239V for Rep, 27S, 28R and 212L for Cap); PCV4b contains 239L for Rep, 27S, 28G and 212L for Cap; PCV4c contains 239V for Rep, 27N, 28R and 212M for Cap [30]. However, two PCV4 reference strains (KU-02010 and KU-02011) from Korea, as well as one reference strain (PCV4-PY) from Henan Province, China, were classified as PCV4a in the phylogenetic tree, yet their Cap protein’s 212th position contained M and P, respectively, instead of L. This indicated that the marker positions suggested by Xu et al. might not be universally applicable. GS2022-BY and GS2022-LZ also contained the essential elements for the replication of circoviruses in pig-origin PCV4 strains predicted by Nguyen et al [34], such as the origin of DNA replication, endonuclease and helicase.

The arginine-rich region known as the nuclear localization signal (NLS) within the circovirus genus plays a role in facilitating the nuclear localization of the viral genome [38–40]. Recently, it was reported that the NLS of PCV4 Cap is located at the N-terminal residues 1–20 [41], which was also observed in GS2022-BY and GS2022-LZ. In the PCV4 Cap, five potential linear B cell epitopes with high antigenicity were predicted by Wang et al [42], including epitope A: 72–88, 104–112, epitope B: 122–177, epitope C:199–205, and epitope D:219–225 (Fig 2). Out of the 50 amino acid mutations found in the Cap protein of the 62 PCV4 strains, 16 were situated within the anticipated epitope region. These mutations might potentially result in a modified antigenic profile of the Cap protein, warranting additional investigation.

## Conclusion

In conclusion, to the best of our knowledge, this study is the first to report the detection of PCV4 DNA in swine farms in Gansu Province with a low positivity rate. Furthermore, we successfully obtained the complete genomes of two PCV4 strains, which were classified into PCV4a and PCV4c clusters. These results enhance our comprehension of the prevalence and genomic characteristics of PCV4 in pig farms located in Gansu Province, China.

## Supporting information

S1 Table List of primer sequences used in this study. (DOCX)

S2 Table The information of all PCV4 strains for sequence alignment and phylogenetic analysis. (DOCX)

## References

[1] Lekcharoensuk P., Morozov I., Paul P. S., Thangthumniyom N., Wajjawalku W. and Meng X. J. Epitope mapping of the major capsid protein of type 2 porcine circovirus (PCV2) by using chimeric PCV1 and PCV2. J Virol. (2004) 78:8135–8145. doi: 10.1128/JVI.78.15.8135-8145.2004 15254185 PMC446101

[2] Cheung A. K. Porcine circovirus: transcription and DNA replication. Virus Res. (2012) 164:46–53. doi: 10.1016/j.virusres.2011.10.012 22036834

[3] Opriessnig T., Karuppannan A. K., Castro Ammg and Xiao C. T. Porcine circoviruses: current status, knowledge gaps and challenges. Virus Res. (2020) 286:198044. doi: 10.1016/j.virusres.2020.198044 32502553

[4] Tischer I, Rasch R and Tochtermann G. Characterization of papovavirus-and picornavirus-like particles in permanent pig kidney cell lines. Zentralblatt fur Bakteriologie, Parasitenkunde, Infektionskrankheiten und Hygiene. Erste Abteilung Originale. Reihe A: Medizinische Mikrobiologie und Parasitologie. (1974) 226:153–167. 4151202

[5] Gm Allan, Mcneilly F Cassidy Jp, Ga Reilly, Adair B Ellis Wa, et al. Pathogenesis of porcine circovirus; experimental infections of colostrum deprived piglets and examination of pig foetal material. Veterinary microbiology. (1995) 44:49–64. doi: 10.1016/0378-1135(94)00136-k 7667906

[6] Tischer I, Mields W, Wolff D, Vagt M and Griem W. Studies on epidemiology and pathogenicity of porcine circovirus. Archives of Virology. (1986) 91:271–276. doi: 10.1007/BF01314286 3778212

[7] Allan Gm, Mcneilly F, Kennedy S, Daft B, Clarke Eg, Ellis Ja, et al. Isolation of porcine circovirus-like viruses from pigs with a wasting disease in the USA and Europe. Journal of veterinary diagnostic investigation: official publication of the American Association of Veterinary Laboratory Diagnosticians, Inc. (1998) 10:3–10. doi: 10.1177/104063879801000102 9526853

[8] Ellis J, Hassard L, Clark E, Harding J, Allan G, Willson P, et al. Isolation of circovirus from lesions of pigs with postweaning multisystemic wasting syndrome. The Canadian veterinary journal = La revue veterinaire canadienne. (1998) 39:44–51. 9442952 PMC1539838

[9] Kiupel M, Stevenson Gw, Mittal Sk, Clark Eg and Haines Dm. Circovirus-like viral associated disease in weaned pigs in Indiana. Veterinary pathology. (1998) 35:303–307. doi: 10.1177/030098589803500411 9684976

[10] Morozov I, Sirinarumitr T, Sorden Sd, Halbur Pg, Morgan Mk, Yoon Kj, et al. Detection of a novel strain of porcine circovirus in pigs with postweaning multisystemic wasting syndrome. Journal of clinical microbiology. (1998) 36:2535–2541. doi: 10.1128/JCM.36.9.2535-2541.1998 9705388 PMC105158

[11] Gp Nayar, Hamel A and Lin L. Detection and characterization of porcine circovirus associated with postweaning multisystemic wasting syndrome in pigs. The Canadian veterinary journal = La revue veterinaire canadienne. (1997) 38:385–386. PMC1576874 9187809

[12] Palinski R., Piñeyro P., Shang P., Yuan F., Guo R., Fang Y., et al. A Novel Porcine Circovirus Distantly Related to Known Circoviruses Is Associated with Porcine Dermatitis and Nephropathy Syndrome and Reproductive Failure. J Virol. (2017) 91: doi: 10.1128/JVI.01879-16 27795441 PMC5165205

[13] Phan T. G., Giannitti F., Rossow S., Marthaler D., Knutson T. P., Li L., et al. Detection of a novel circovirus PCV3 in pigs with cardiac and multi-systemic inflammation. Virol J. (2016) 13:184. doi: 10.1186/s12985-016-0642-z 27835942 PMC5105309

[14] Zhang H. H., Hu W. Q., Li J. Y., Liu T. N., Zhou J. Y., Opriessnig T., et al. Novel circovirus species identified in farmed pigs designated as Porcine circovirus 4, Hunan province, China. Transbound Emerg Dis. (2020) 67:1057–1061. doi: 10.1111/tbed.13446 31823481

[15] Zhang D., Bai C., Ge K., Li Y., Gao W., Jiang S., et al. Establishment of an SYBR Green-based real-time PCR assay for porcine circovirus type 4 detection. J Virol Methods. (2020) 285:113963. doi: 10.1016/j.jviromet.2020.113963 32882322

[16] Chen N., Xiao Y., Li X., Li S., Xie N., Yan X., et al. Development and application of a quadruplex real-time PCR assay for differential detection of porcine circoviruses (PCV1 to PCV4) in Jiangsu province of China from 2016 to 2020. Transbound Emerg Dis. (2021) 68:1615–1624. doi: 10.1111/tbed.13833 32931644

[17] Sun W., Du Q., Han Z., Bi J., Lan T., Wang W., et al. Detection and genetic characterization of porcine circovirus 4 (PCV4) in Guangxi, China. Gene. (2021) 773:145384. doi: 10.1016/j.gene.2020.145384 33383119

[18] Kim D. Y., Kim H. R., Park J. H., Kwon N. Y., Kim J. M., Kim J. K., et al. Detection of a novel porcine circovirus 4 in Korean pig herds using a loop-mediated isothermal amplification assay. J Virol Methods. (2022) 299:114350. doi: 10.1016/j.jviromet.2021.114350 34748817

[19] Xu T., Hou C. Y., Zhang Y. H., Li H. X., Chen X. M., Pan J. J., et al. Simultaneous detection and genetic characterization of porcine circovirus 2 and 4 in Henan province of China. Gene. (2022) 808:145991. doi: 10.1016/j.gene.2021.145991 34626723

[20] Tian R. B., Zhao Y., Cui J. T., Zheng H. H., Xu T., Hou C. Y., et al. Molecular detection and phylogenetic analysis of Porcine circovirus 4 in Henan and Shanxi Provinces of China. Transbound Emerg Dis. (2021) 68:276–282. doi: 10.1111/tbed.13714 32634296

[21] Vargas-Bermudez D. S., Mogollón J. D. and Jaime J. The Prevalence and Genetic Diversity of PCV3 and PCV2 in Colombia and PCV4 Survey during 2015–2016 and 2018–2019. Pathogens. (2022) 11: doi: 10.3390/pathogens11060633 35745487 PMC9228467

[22] Franzo G., Ruiz A., Grassi L., Sibila M., Drigo M. and Segalés J. Lack of Porcine circovirus 4 Genome Detection in Pig Samples from Italy and Spain. Pathogens. (2020) 9: doi: 10.3390/pathogens9060433 32486429 PMC7350368

[23] Niu G., Zhang X., Ji W., Chen S., Li X., Yang L., et al. Porcine circovirus 4 rescued from an infectious clone is replicable and pathogenic in vivo. Transbound Emerg Dis. (2022) 69:e1632–e1641. doi: 10.1111/tbed.14498 35240007

[24] Zhao Y., Han H. Y., Fan L., Tian R. B., Cui J. T., Li J. Y., et al. Development of a TB green II-based duplex real-time fluorescence quantitative PCR assay for the simultaneous detection of porcine circovirus 2 and 3. Mol Cell Probes. (2019) 45:31–36. doi: 10.1016/j.mcp.2019.04.001 30980890

[25] Wang X. Y., Ji C. J., Zhang X., Xu D. P. and Zhang D. L. Infection, genetic and virulence characteristics of porcine epidemic diarrhea virus in northwest China. Infect Genet Evol. (2018) 62:34–39. doi: 10.1016/j.meegid.2018.04.001 29625238

[26] Zhou L., Sun Y., Lan T., Wu R., Chen J., Wu Z., et al. Retrospective detection and phylogenetic analysis of swine acute diarrhoea syndrome coronavirus in pigs in southern China. Transbound Emerg Dis. (2019) 66:687–695. doi: 10.1111/tbed.13008 30171801 PMC7168530

[27] Tian R. B., Jin Y., Xu T., Zhao Y., Wang Z. Y. and Chen H. Y. Development of a SYBR green I-based duplex real-time PCR assay for detection of pseudorabies virus and porcine circovirus 3. Mol Cell Probes. (2020) 53:101593. doi: 10.1016/j.mcp.2020.101593 32387303

[28] Zheng L. L., Chai L. Y., Tian R. B., Zhao Y., Chen H. Y. and Wang Z. Y. Simultaneous detection of porcine reproductive and respiratory syndrome virus and porcine circovirus 3 by SYBR Green І-based duplex real-time PCR. Mol Cell Probes. (2020) 49:101474. 10.1016/j.mcp.2019.101474 31655106

[29] Kim O., Choi C., Kim B. and Chae C. Detection and differentiation of porcine epidemic diarrhoea virus and transmissible gastroenteritis virus in clinical samples by multiplex RT-PCR. Vet Rec. (2000) 146:637–640. doi: 10.1136/vr.146.22.637 10872784

[30] Xu T., Chen X. M., Fu Y., Ai Y., Wang D. M., Wei Z. Y., et al. Cross-species transmission of an emerging porcine circovirus (PCV4): First molecular detection and retrospective investigation in dairy cows. Vet Microbiol. (2022) 273:109528. doi: 10.1016/j.vetmic.2022.109528 35944390

[31] Ha Z., Yu C., Xie C., Wang G., Zhang Y., Hao P., et al. Retrospective surveillance of porcine circovirus 4 in pigs in Inner Mongolia, China, from 2016 to 2018. Arch Virol. (2021) 166:1951–1959. doi: 10.1007/s00705-021-05088-w 33987752

[32] Hou C. Y., Zhang L. H., Zhang Y. H., Cui J. T., Zhao L., Zheng L. L., et al. Phylogenetic analysis of porcine circovirus 4 in Henan Province of China: A retrospective study from 2011 to 2021. Transbound Emerg Dis. (2022) 69:1890–1901. doi: 10.1111/tbed.14172 34076964

[33] Nguyen V. G., Do H. Q., Huynh T. M., Park Y. H., Park B. K. and Chung H. C. Molecular-based detection, genetic characterization and phylogenetic analysis of porcine circovirus 4 from Korean domestic swine farms. Transbound Emerg Dis. (2022) 69:538–548. doi: 10.1111/tbed.14017 33529468

[34] Zhang L. H., Wang T. X., Fu P. F., Zhao Y. Y., Li H. X., Wang D. M., et al. First Molecular Detection and Genetic Analysis of a Novel Porcine Circovirus (Porcine Circovirus 4) in Dogs in the World. Microbiol Spectr. (2023) e0433322. doi: 10.1128/spectrum.04333-22 36728419 PMC10100769

[35] Wang Y., Yan S., Ji Y., Yang Y., Rui P., Ma Z., et al. First Identification and Phylogenetic Analysis of Porcine Circovirus Type 4 in Fur Animals in Hebei, China. Animals (Basel). (2022) 12: doi: 10.3390/ani12233325 36496846 PMC9737481

[36] Wu H., Hou C., Wang Z., Meng P., Chen H. and Cao H. First complete genomic sequence analysis of porcine circovirus type 4 (PCV4) in wild boars. Vet Microbiol. (2022) 273:109547. doi: 10.1016/j.vetmic.2022.109547 36037620

[37] Franzo G., Delwart E., Fux R., Hause B., Su S., Zhou J., et al. Genotyping Porcine Circovirus 3 (PCV-3) Nowadays: Does It Make Sense? Viruses. (2020) 12: doi: 10.3390/v12030265 32121102 PMC7150946

[38] Liu Q., Tikoo S. K. and Babiuk L. A. Nuclear localization of the ORF2 protein encoded by porcine circovirus type 2. Virology. (2001) 285:91–99. doi: 10.1006/viro.2001.0922 11414809

[39] Shuai J., Wei W., Jiang L., Li X., Chen N. and Fang W. Mapping of the nuclear localization signals in open reading frame 2 protein from porcine circovirus type 1. Acta Biochim Biophys Sin (Shanghai). (2008) 40:71–77. doi: 10.1111/j.1745-7270.2008.00377.x 18180855

[40] Mou C., Wang M., Pan S. and Chen Z. Identification of Nuclear Localization Signals in the ORF2 Protein of Porcine Circovirus Type 3. Viruses. (2019) 11: doi: 10.3390/v11121086 31766638 PMC6950156

[41] Zhou J., Qiu Y., Zhu N., Zhou L., Dai B., Feng X., et al. The Nucleolar Localization Signal of Porcine Circovirus Type 4 Capsid Protein Is Essential for Interaction With Serine-48 Residue of Nucleolar Phosphoprotein Nucleophosmin-1. Front Microbiol. (2021) 12:751382. doi: 10.3389/fmicb.2021.751382 34745055 PMC8566881

[42] Wang D., Mai J., Lei B., Zhang Y., Yang Y. and Wang N. Structure, Antigenic Properties, and Highly Efficient Assembly of PCV4 Capsid Protein. Front Vet Sci. (2021) 8:695466. doi: 10.3389/fvets.2021.695466 34504886 PMC8421537

