# Supplementary material for: First molecular detection and genetic characterization of porcine circovirus 4 in the Gansu Province of China
Source: PLoS One. 2024 Feb 5;19(2):e0293135. doi: 10.1371/journal.pone.0293135 (PMC10843115; doi:10.1371/journal.pone.0293135)
Supplement: S1 Table — (DOCX) [file pone.0293135.s001.docx]

S1 Table List of primer sequences used in this study

| Primer name | Nucleotide sequence (5’-3’) | Primer locations (bp) | Product size |
| --- | --- | --- | --- |
| PCV4-1F | GAGGTTCCACCCGTTTAAG | 260-278 | 577 |
| PCV4-1R | CCAGTCCTTGATCTGCTTGTTG | 815-836 |  |
| PCV4-2F | GCCAAGACAATGTGGATTACC | 792-812 | 690 |
| PCV4-2R | AGCCTCCCATTTGCATATTACC | 1460-1481 |  |
| PCV4-3F | CCACATAGTCTCCATCCAGTTG | 1361-1382 | 769 |
| PCV4-3R | CCCTCCTTTGGAGCAATACTT | 339-359 |  |
