# Supplementary material for: First molecular detection and genetic characterization of porcine circovirus 4 in the Gansu Province of China
Source: PLoS One. 2024 Feb 5;19(2):e0293135. doi: 10.1371/journal.pone.0293135 (PMC10843115; doi:10.1371/journal.pone.0293135)
Supplement: S2 Table — (DOCX) [file pone.0293135.s002.docx]

| Strain | Host | Size | Collection date | Country | Accession number | Strain | Host | Size | Collection date | Country | Accession number |
| --- | --- | --- | --- | --- | --- | --- | --- | --- | --- | --- | --- |
| HNU-AHG1-2019 | domestic pig | 1770 | Feb-2019 | China | MK986820.1 | HN-ZK-201707 | pig | 1770 | Jul-2017 | China | MW600960.1 |
| Henan-LY1-2019 | domestic pig | 1770 | Feb-2019 | China | MT015686.1 | HN-LY-202005 | pig | 1770 | May-2020 | China | MW538943.1 |
| KF-02-2019 | domestic pig | 1770 | Oct-2019 | China | MT193105.1 | HN-LY-202006 | pig | 1770 | Jun-2020 | China | MW600947.1 |
| KF-01-2019 | domestic pig | 1770 | Oct-2019 | China | MT193106.1 | HN-LY-202007 | pig | 1770 | Jul-2020 | China | MW600948.1 |
| PCV4/GX2020/NN88 | domestic pig | 1770 | 2018 | China | MT311852.1 | HN-SMX-202011 | pig | 1770 | Nov-2020 | China | MW600949.1 |
| PCV4/GX2020/GL69 | domestic pig | 1770 | 2018 | China | MT311853.1 | HN-XX-201811 | pig | 1770 | Nov-2018 | China | MW600950.1 |
| PCV4/GX2020/FCG49 | domestic pig | 1770 | 2018 | China | MT311854.1 | HN-KF-201812 | pig | 1770 | Dec-2018 | China | MW600951.1 |
| FJ-PCV4 | domestic pig | 1770 | 2019 | China | MT721742.1 | HN-HB-201704 | pig | 1770 | Apr-2017 | China | MW600952.1 |
| JSYZ1901-2 | domestic pig | 1770 | 02-Jan-2019 | China | MT769268.1 | HN-XX-201212 | pig | 1770 | Dec-2012 | China | MW600953.1 |
| E115 | domestic pig | 1770 | 23-Apr-2020 | South Korea | MT882344.1 | HN-LY-201702 | pig | 1770 | Feb-2017 | China | MW600954.1 |
| PCV4/CN/NM1/2017 | domestic pig | 1770 | 2017 | China | MT882410.1 | HN-ZZ-201603 | pig | 1770 | Mar-2016 | China | MW600955.1 |
| PCV4/CN/NM2/2017 | domestic pig | 1770 | 2017 | China | MT882411.1 | HN-ZK-201512 | pig | 1770 | Dec-2015 | China | MW600956.1 |
| PCV4/CN/NM3/2017 | domestic pig | 1770 | 2017 | China | MT882412.1 | HN-ZK-201601 | pig | 1770 | Jan-2016 | China | MW600957.1 |
| Hebei-AP1-2019 | domestic pig | 1770 | 2019 | China | MW084633.1 | HN-ZMD-201212 | pig | 1770 | Dec-2012 | China | MW600958.1 |
| Hebei1 | domestic pig | 1770 | 10-Sep-2020 | China | MW262973.1 | HN-XX-201601 | pig | 1770 | Jan-2016 | China | MW600959.1 |
| Hebei2 | domestic pig | 1770 | 15-Sep-2020 | China | MW262974.1 | KU-02011 | pig | 1770 | Nov-2020 | South Korea | MW712667.1 |
| Hebei3 | domestic pig | 1770 | 15-Sep-2020 | China | MW262975.1 | KU-02010 | pig | 1770 | Oct-2020 | South Korea | MW712668.1 |
| Hebei4 | domestic pig | 1770 | 20-Sep-2020 | China | MW262976.1 | PCV4-YY2019 | pig | 1770 | 2019 | China | MW759027.1 |
| Hebei5 | domestic pig | 1770 | 20-Sep-2020 | China | MW262977.1 | PCV4-HB2017 | pig | 1770 | 2017 | China | MW759028.1 |
| Hebei6 | domestic pig | 1770 | 20-Sep-2020 | China | MW262978.1 | PCV4-LY2017 | pig | 1770 | 2017 | China | MW759029.1 |
| Hebei-Rac1 | raccoon dog | 1770 | 01-Oct-2015 | China | MW262979.1 | JXWY-2021 | wild boar | 1770 | 2020 | China | MW988108.1 |
| Hebei-Rac2 | raccoon dog | 1770 | 07-Nov-2017 | China | MW262980.1 | JXSC-2021 | wild boar | 1770 | 2020 | China | MW988109.1 |
| Hebei-Rac3 | raccoon dog | 1770 | 16-Jun-2019 | China | MW262981.1 | SC-GA2022ABTC | pig | 1770 | 2022 | China | OP497960.1 |
| Hebei-Rac4 | raccoon dog | 1770 | 13-Jun-2018 | China | MW262982.1 | PCV4-LY2020 | pig | 1770 | 2020 | China | MW759026.1 |
| Hebei-Rac5 | raccoon dog | 1770 | 02-Jun-2018 | China | MW262983.1 | FJ2020001 | pig | 1770 | Jan-2020 | China | MW238796.1 |
| Hebei-Fox1 | fox | 1770 | 25-Jun-2018 | China | MW262984.1 | SCABTC-Dog2022 | pig | 1770 | 2022 | China | OP948894.1 |
| PCV4-PY | pig | 1770 | 2021 | China | MZ593770.1 | PCV4-HB | pig | 1770 | 2021 | China | MZ593769.1 |
| PCV4-XX | pig | 1770 | 2021 | China | MZ593773.1 | ZZ2020-DC | pig | 1770 | 04-Nov-2020 | China | ON470198.1 |
| PCV4-XC2 | pig | 1770 | 2021 | China | MZ593772.1 | NY2012-DC | pig | 1770 | 11-Jun-2012 | China | ON470197.1 |
| PCV4-XC1 | pig | 1770 | 2021 | China | MZ593771.1 | GS2022-BY* | pig | 1770 | 16-Oct-2022 | China: Gansu | OQ970016.1 |
| LY2019-DC | pig | 1770 | 23-Apr-2019 | China | ON470196.1 | GS2022-LZ* | pig | 1770 | 13-Dec-2022 | China: Gansu | OQ970017.1 |

S2 Table The information of all PCV4 strains for sequence alignment and phylogenetic analysis
